# HIV/AIDS, Comorbidity, and Alcohol: Can We Make a Difference?

**Published:** 2010

**Authors:** Amy Justice, Lynn Sullivan, David Fiellin, for the Veterans Aging Cohort Study Project Team

**Keywords:** Alcohol consumption, human immunodeficiency virus, acquired immune deficiency syndrome, comorbidity, disease factor, aging, Veterans Aging Cohort study, treatment method

## Abstract

Alcohol use is common among people infected with HIV and plays an important role in their health outcomes. Because alcohol use complicates HIV infection and contributes to comorbid diseases, it is important for researchers and practitioners to understand these interactions and to integrate alcohol treatment with medical management of long-term HIV infection and associated comorbidity. The Veterans Aging Cohort Study (VACS) is a large, multisite study of the effects of alcohol use on HIV outcomes in the broader context of aging. A multilevel strategy intervention trial is needed to address the many modifiable implications of alcohol consumption among those receiving treatment for HIV.

Alcohol use is common among people at risk for, and aging with, human immunodeficiency virus (HIV) infection and plays a central modifiable role in their health outcomes (Braithwaite et al. 2007; Conigliaro et al. 2003, 2004; Cooper and Cameron 2005; Justice et al. 2004, 2006*b*; McGinnis et al. 2006; Rees et al. 2001; Samet et al. 2004; Shaffer et al. 2004). Past and present alcohol consumption directly influences HIV progression and survival by altering timing of and adherence and response to medication designed to minimize levels of HIV in the body (i.e., antiretroviral treatment [ART]) (Bean 2000; Braithwaite et al. 2005, 2007, 2008; Cook et al. 2006; Kresina et al. 2002; Samet et al. 1998, 2003). Alcohol use also influences patient outcomes by increasing the risk for HIV- and antiretroviral-associated comorbidities, including liver disease, cardiovascular and cerebrovascular disease, pulmonary disease, bone disease, and cancer (Conigliaro et al. 2003, 2006; Justice et al. 2006*b*). People with HIV have a lower tolerance for alcohol (Braithwaite et al. 2008) yet maintain heavy levels of consumption as they age (Green 2009). The cumulative effects of past and current alcohol consumption is likely to increase now that patients with HIV infection are expected to live 20 to 30 years on ART (Hogg et al. 2008).

Health care providers can help to mitigate the harmful effects of alcohol use in patients with HIV. A growing body of research has demonstrated that behavioral and pharmacologic interventions for alcohol can be implemented successfully in primary-care and office-based settings (see Samet and Walley, pp. 267–279 in this issue). However, practitioners and researchers must adapt and coordinate such interventions to the complex clinical context of HIV infection. This will require prioritization and integration of alcohol treatment with medical management of long-term HIV infection and associated comorbidity. The only reasonable way to integrate and jointly prioritize treatment for HIV, comorbid disease, and alcohol use is to estimate the impact each condition has on the patient’s risk of morbidity and mortality and thus help inform patient and provider decisionmaking (Braithwaite et al. 2007; Justice 2006). Researchers must therefore develop methods to prioritize, integrate, and coordinate treatment for alcohol, HIV, and associated comorbid conditions.

Because HIV infection has become a complex chronic disease in which alcohol has a multifaceted impact on health outcomes, strategy implementation studies are needed. Strategy implementation studies combine behavioral and pharmacologic methods to decrease alcohol consumption with clinical strategies to mitigate the short-and long-term effects of alcohol on morbidity and mortality. Long-term (i.e., longitudinal) observational studies are particularly useful in helping to characterize HIV-infected populations at risk and their alcohol-associated disease trajectories. Further, data from observational studies can suggest means of objectively gauging the effects of this combined approach. This article reviews the prevalence of alcohol use among people with HIV and the complex and interacting role of alcohol use in HIV and selected comorbid diseases, describes ongoing plans for continued longitudinal observation, and, finally, discusses the authors’ plan to develop multilevel strategy implementation trials within the Veterans Aging Cohort Study (VACS).

## Prevalence

The majority of people receiving care for HIV infection report current alcohol consumption (i.e., consuming alcohol during the previous 12 months). Among people coinfected with hepatitis C virus (HCV) or with evidence of liver injury, the proportion comprising current alcohol users is even higher (Conigliaro et al. 2006; Goulet et al. 2005). In a national sample of patients with HIV, 8 to 12 percent were classified as current heavy drinkers, a rate approximately twice that of the U.S. national average (Burnam et al. 2001; Galvan et al. 2003). The lifetime prevalence of alcohol use disorders in patients with HIV is two to three times that of the general population (Conigliaro et al. 2006; Connors and Volk 2003; Isaacson and Schorling 1999; O’Connor and Schottenfeld 1998; Reid et al. 1999, 2002). Finally, in contrast to findings from populations of people without HIV, there is no evidence of a protective effect from alcohol in people with HIV (Ellison 2002).

## Alcohol and Medication Nonadherence

Alcohol problems in HIV-infected patients are associated with poor adherence to combination antiretroviral therapy (CART) medications (Braithwaite et al. 2005, 2007, 2008; Cook et al. 2001). The VACS, a large National Institute on Alcohol Abuse and Alcoholism (NIAAA)-funded national sample of HIV-infected and HIV-negative patients (Justice et al. 2006*a*), examined the relationship between alcohol consumption and medication adherence and found that adherence was lower on days on which patients drank heavily and on the following day. The effect of alcohol was more pronounced in HIV-infected individuals; non–binge drinkers were 1.8 times more likely to have lower adherence, and binge drinkers were 4.3 times more likely to have lower adherence (Braithwaite et al. 2005). Further, alcohol consumption was the most significant predictor of CART medication adherence, with the greatest adherence associated with recent abstinence from alcohol. A separate study noted that adjusting alcohol quantity for level of average self-reported consumption and self-reported threshold for intoxication improves the association between alcohol and nonadherence (Braithwaite et al. 2008; Justice et al. 2006*a*). Heavy drinking in HIV-infected patients also is associated with poor treatment response, as evidenced by lower counts of immune cells (i.e., CD4 lymphocytes) and higher HIV RNA (Samet et al. 2003). In turn, it has been shown that people who stop drinking have an improved response to HIV therapy (Samet et al. 2003).

## Alcohol and Antiretroviral Resistance

Ongoing heavy drinking is strongly associated with poor CART adherence. Suboptimal CART adherence not only leads to HIV progression but also to antiretroviral resistance (Braithwaite et al. 2006, 2007). HIV drug resistance results from mutations that arise in the viral proteins targeted by antiretroviral medications. Antiretroviral resistance diminishes the effectiveness of treatment for individual patients and for individuals subsequently infected with these viral strains. Mutations can cause resistance to a single medication or an entire class of antiretrovirals. HIV drug resistance can lead to an inability of the drug or class of drugs to suppress the HIV virus and, therefore, limits future treatment options.

## Alcohol, Risky Sexual Behavior, and HIV Transmission

Alcohol use also is strongly associated with risky sexual behavior among people with HIV infection (Cook et al. 2006; Leigh 1990; Rees et al. 2001; Stein et al. 2005). Because people using alcohol are less likely to adhere to their antiretroviral medication, they are more likely to have a high virus load, including resistant virus. For instance, in one study of 168 people with viral resistance, frequent alcohol use was associated with unprotected intercourse with HIV-infected partners (Chin-Hong et al. 2005). Therefore, people with HIV who consume significant amounts of alcohol pose a particularly high risk of HIV transmission, and the virus transmitted already may be resistant to at least some forms of ART.

## HIV, Aging, and Alcohol

Many studies have found that people with HIV experience age-related comorbid disease, organ system functional decline, and frailty at an earlier age than demographically similar control subjects (Appay et al. 2007; Bestilny et al. 2000; Desquilbet et al. 2007; Effros 2004; Oursler et al. 2006). This is likely to be accentuated among those consuming harmful amounts of alcohol. Lifetime patterns of substance abuse differ for people with HIV infection compared with demographically similar uninfected individuals (Green 2009). Specifically, patterns of heavy consumption continue into middle and older ages and are paralleled by similar patterns of drug and tobacco use. Yet people with HIV infection have lower tolerance for alcohol (Braithwaite et al. 2008).

Much of the continuing morbidity among patients receiving CART has been attributed to chronic inflammation resulting from the depletion of tissue important in immune cell generation (i.e., lymphoid tissue). This depletion causes increased intestinal permeability (Deeks 2009; Deeks and Phillips 2009; Kuller et al. 2008; Weber et al. 2006), which also can occur with alcohol use, leading to similar medical consequences (Purohit et al. 2008). Taken together, these observations suggest a larger harmful effect of alcohol among middle-aged and older individuals with HIV infection than among demographically similar uninfected control subjects (see figure 1).

Similarly, a large proportion of people with HIV infection who are not currently drinking have a substantial history of alcohol abuse and dependence and are more likely to report that they stopped drinking because they were too ill to continue. As a result, many HIV patients experience alcohol-related medical consequences even though they may no longer be drinking (Justice et al. 2006*b*). Providers often fail to detect current alcohol consumption; they almost never ask about past consumption (Conigliaro et al. 2003). As a result, they are unlikely to detect organ system injury resulting from prior alcohol consumption (Corrao et al. 2004).

## HIV, Alcohol, and Comorbid Disease

HIV and alcohol use are associated with increased risk for various types of disease. Some of these comorbidities are described below.

### Liver Disease

HIV infection and particular ARTs substantially increase the risk for incidence and progression of liver disease (Sulkowski 2008*a*, *b*; Sulkowski et al. 2007). Even low levels of liver injury are associated with substantial increases in risk of mortality among patients with HIV infection (Justice et al. 2009). These levels of injury are common among HIV-infected patients receiving care due to multiple causes, including alcohol use viral hepatitis, ART toxicity, and, likely, nonantiretroviral medication toxicity. Morbidity and mortality associated with alcohol-induced liver injury is well described and is marked by progressive liver inflammation and the formation of scar tissue (i.e., fibrosis), leading to cirrhosis and its complications. Among people with chronic HCV infection, regular alcohol use contributes to an increased risk of cirrhosis, inability of the liver to recover from damage (i.e., liver decompensation), cancer, and death (Deuffic-Burban et al. 2007; Sulkowski 2008*a*, *c*). Although liver cell injury is increasingly recognized as a particularly important source of morbidity and mortality among people with HIV, it is largely ascribed to coexisting viral hepatitis and medication-associated liver damage (e.g., antiretroviral drugs). The independent impact of varying and even low levels of alcohol consumption on progression of this injury has not always been clearly identified. Lim and colleagues (2008) recently noted that liver injury and fibrosis increase directly with increasing levels of alcohol consumption. Significant increases in advanced fibrosis or cirrhosis are found at all levels of alcohol exposure among people with HIV only (8.6 percent), HCV only (13.8 percent), and HCV–HIV coinfection (31.8 percent). In multivariate analysis, after controlling for HCV and HIV infections, alcohol abuse or dependence represented the strongest predictor of advanced fibrosis (Lim et al. 2008). Based on evidence that even low levels of alcohol can act synergistically with HIV, HCV, and CART to cause hepatoxicity, strategies that can decrease the adverse impact of alcohol, such as effective treatments for alcohol consumption, are likely to yield substantial benefits.

### Cardiovascular and Cerebrovascular Disease

HIV and ART have been associated with increased risk of both cardiovascular and cerebrovascular disease (De Lorenzo et al. 2008; Freiberg et al. 2007; Justice et al. 2008). Heavy alcohol consumption is associated with both of these conditions through multiple mechanisms. Heavy alcohol use leads to adverse effects on blood pressure (McFadden et al. 2005) and cardiac muscle function (Djousse and Gaziano 2008) and contributes to the risk of irregular heart rate (Balbao et al. 2009; Conen et al. 2008). A systematic review (McFadden et al. 2005) on the effect of recent alcohol consumption on blood pressure found a significant rise in systolic blood pressure and diastolic blood pressure of 2.7 and 1.4 mmHg, respectively, following daily alcohol ingestion. Consumption of at least three drinks per day is associated with greater risk for hemorrhagic stroke, and greater alcohol consumption also may increase risk for ischemic stroke (Donahue et al. 1986; Iso et al. 2004; Kiyohara et al. 1995).

### Pulmonary Disease

Even after adjusting for smoking exposure, risk of bacterial pneumonia and chronic obstructive pulmonary disease are elevated among people with HIV compared with uninfected control subjects (Crothers et al. 2006; Justice et al. 2006*b*; Rodriguez-Barradas et al. 2008). Heavy alcohol use also increases risk for pulmonary disease. Alcohol affects both innate and adaptive immune responses, leading to immunosuppression (Szabo and Mandrekar 2009). This helps to explain the relationship between chronic alcohol consumption and an increased incidence of bacterial and viral pneumonias (Happel and Nelson 2005). In addition, research shows an increased association between alcohol and acute and chronic lung disease, especially chronic obstructive pulmonary disease (Guidot and Hart 2005; Joshi and Guidot 2007).

### Bone Disease

Recent work has demonstrated that people with HIV have lower bone density (i.e., osteoporosis) (Brown and Qaqish 2006) than control subjects and may have a greater risk of fracture (Brown and Qaqish 2006; Triant et al. 2008). Both alcohol and cigarette smoking (a common comorbid behavior among drinkers) increase this risk. Alcohol consumption disrupts bone remodeling by suppressing new bone formation and causing increases in bone resorption (Chakkalakal 2005). The resulting bone loss means that people who are alcohol dependent frequently have low bone mass, decreased bone formation, and an increased rate of fractures. Alcohol consumption is a risk factor for osteoporosis, especially at higher levels of consumption. A recent review (Berg et al. 2008) found that compared with abstainers, people consuming more than 0.5 to 1.0 alcoholic drinks per day had lower hip fracture risk but people consuming more than 2 alcoholic drinks per day had a higher risk of hip fracture.

### Cancer

HIV infection is associated with an increased risk for many common cancers (anal, cervical, liver, colon, lung, and leukemia), not all of which are associated with viral infections (e.g., anal, cervical, and liver cancer) (Bedimo et al. 2009; Patel et al. 2008). Increased alcohol consumption has been associated with cancers of the digestive tract (e.g., mouth, pharynx, larynx, esophagus), upper airway, breast, and liver (Corrao et al. 2004). A recent meta-analysis (Bagnardi et al. 2001) was not able to determine a level of alcohol consumption below which no risk for cancer was evident. In addition to the malignancies noted above, researchers found increases in the risk for cancers of the stomach, colon, rectum, and ovaries. The meta-analysis was able to document that study participants who consumed four drinks per day had an increased risk for cancer at multiple sites.

## The Role of Longitudinal Observational Analyses Such As VACS

The review above highlights specific areas in which data on the association between alcohol and multiple medical conditions exist. Nonetheless, the existing literature often is limited by its cross-sectional nature, limited follow-up, or limited information regarding alcohol consumption and alcohol-related disorders. Therefore, long-term observational studies, including study participants with and without HIV, can play an essential role in providing information about the role of alcohol in HIV and comorbid medical conditions. These studies may help elucidate the impact of alcohol consumption and other behaviors on the progression of common medical conditions. The advantage of observational methods is that they provide information about the natural history of these conditions and the real-world efficacy of standard treatments. Whereas other research designs such as randomized clinical trials can provide information on treatment efficacy, observational studies provide unique information regarding causal associations, prognosis, risk, and the effect of treatment in populations that are more diverse than those often included in clinical trials. In this way, observational methods can provide unique information that can be used to inform intervention studies designed to maximize treatment strategies and target risk factors and exposures.

### What Is VACS?

The VACS is a study of HIV-positive patients attending infectious disease clinics and age-, race-, and site-matched HIV-uninfected patients in general medicine clinics (Justice et al. 2006*a*). VACS is a multisite, multiwave study being conducted at eight Veterans Health Administration (VA) health care facilities: Atlanta, GA; Baltimore, MD; Bronx, NY; Houston, TX; Los Angeles, CA, New York City, NY; Pittsburgh, PA; and Washington, DC. To date, VACS is the largest alcohol-focused (approximately 7,000 participants) study of its kind with the longest follow-up (currently 7 to 8 years). The primary aim of VACS is to explore the effects of alcohol on HIV outcomes in the broader context of aging. Since 2002, VACS has collected annual self-reported surveys, electronic medical records, and pharmacy data on all participants. Based on information collected in VACS, and recent advances in the field of HIV research, a number of cogent clinical questions regarding alcohol and aging have emerged. In the sections that follow, some of these important unanswered questions are outlined.

#### How do the long-term effects of alcohol differ among HIV-positive and HIV-negative populations?

Long-term observational studies help characterize the trajectory of risk associated with varying levels of alcohol exposure (at risk, binge, abuse/dependence) on HIV and comorbid medical conditions. For example, the impact of an intervention to improve outcomes among those with early liver injury (a common problem among people with HIV and one clearly associated with alcohol exposure) would need to be based on the expected trajectory of liver injury without intervention. Without information on the natural history of disease processes, it is impossible to determine eligibility criteria or the appropriate size of the study population, estimate potential effect sizes, stipulate cogent outcomes, or specify the appropriate follow-up intervals. Long-term studies of alcohol- and HIV-associated comorbid conditions such as liver disease, cardiovascular disease, pulmonary disease, and cancer may require a minimum of 10 to 15 years of observation to allow accurate characterization of the trajectory of clinically relevant events. This is particularly important in studying the relative risk of these diseases seen in HIV-infected patients (compared with HIV-negative control subjects) and with patients receiving CART treatment for two reasons: (1) the relative risk for these events in HIV-infected patients can only be accurately measured after adjusting for increased competing risk of mortality (Chiang 1991) and (2) the relative risk of treatment-associated comorbidity in HIV-infected patients for diseases such as diabetes, high cholesterol, and obesity increases after CART initiation.

#### How does alcohol affect the timing of initiating CART and the choice of medication regimens?

CART regimens have evolved since their initial introduction. In addition, as more data is acquired, consensus regarding the optimal time in the disease process (e.g., CD4 cell count) to initiate ART has evolved. There are four important changes in CART that have occurred which may impact its interactions with alcohol. First, regimens are substantially simpler than in the past, now requiring only one to two doses a day. Because simple regimens are associated with improved adherence, this may mitigate the negative association between alcohol and adherence. Second, regimens appear to have fewer toxic effects, but this remains to be demonstrated in patients who consume substantial amounts of alcohol. Third, particular regimens have been shown to be active against chronic hepatitis B virus (HBV) (the effects of which are magnified among those who actively consume alcohol), but it remains unknown whether antiretroviral activity is maintained over time or overcome by rapid resistance. Finally, it remains to be determined whether people with substance use should start receiving CART after they are engaged in substance abuse treatment or as soon as possible with concomitant management of their substance use.

#### Are there specific populations who are not reaping all the benefits of the CART era?

The landscape of HIV infection in the United States continues to evolve due to the widespread use of CART. The burden of HIV infection is differentially borne by those who are aging and, increasingly, African American men and women (Centers for Disease Control and Prevention 2008; Hall et al. 2009). HIV infection now is viewed more as a chronic medical condition than a rapidly fatal illness. With this in mind it is useful to think of alcohol-associated comorbid medical conditions as increasingly contributing to the morbidity and mortality of people with HIV. For example, liver disease is a leading cause of death among patients receiving treatment for HIV infection. HIV and HBV and/or HCV coinfection is a growing problem in HIV management and one that is clearly exacerbated by alcohol use (Deuffic-Burban et al. 2007; Sulkowski 2008*c*; Sulkowski et al. 2007). In addition, liver cancer, one of the end-stage complications of HCV infection and alcohol-related liver disease, is on the rise (Deuffic-Burban 2007; Sulkowski et al. 2007). Despite this, only a handful of patients with chronic HCV receive appropriate pharmacologic treatment (e.g., interferon, ribavirn) for this disease, and less than half of those who do have a sustained hepatitis C viral response (Butt et al. 2006; Fultz et al. 2003).

#### What is causing the observed differences in morbidity and mortality among people with HIV who consume alcohol?

As differences in HIV disease progression, end-organ injury, and mortality associated with alcohol exposure are characterized in the CART era, it is important to identify modifiable causative factors that can serve as targets for intervention. Current hypotheses regarding the mechanisms by which alcohol adversely affects morbidity and mortality include primary effects on medication adherence and viral resistance and indirect effects on microbial translocation, chronic inflammation, and decreased immune function (Braithwaite et al. 2005, 2008; Brenchley and Douek 2007; Butt et al. 2009; De et al. 2007; Douek 2007; Effros et al. 2008; Freiberg et al. 2007; Kozal 2009; Simen et al. 2009). The only way to further our understanding of these mechanisms is to collect longitudinal measurements of exposures, adherence, and serial tissue samples for ongoing translational research in which biochemical, substances that can be measured, are used to track disease (i.e., biomarkers) may be evaluated in light of a well-characterized clinical history. This will entail large-scale longitudinal sampling in important subpopulations including aging individuals, people of color, people with HCV infection, and people newly initiating CART.

#### What measures are available to explicate the influence of alcohol on overall health in people with HIV infection?

A major difficulty in alcohol research is the lack of a gold-standard measurement for exposure. Alcohol levels in the blood stream reflect only recent exposure, and other biomarkers that reflect longer-term exposure are not sensitive or specific (Allen et al. 2003; Fiellin et al. 2000; Figlie et al. 2002). As a result, intervention trials have relied on patient self-report of alcohol consumption, most often measured in drinks per day (Maisto et al. 1995).

Although most studies have demonstrated that alcohol interventions can affect patient reports of drinks per day over short and intermediate terms (1 to 12 months), few have documented a change in morbidity or mortality. Despite research demonstrating that shorter self-report measures perform as well as longer, more involved surveys (Gordon et al. 2001; Maisto et al. 2008), concerns about substantial reporting bias remain. For example, control groups demonstrate decreasing reports of drinks per day over the course of most alcohol intervention studies. Therefore, it remains unclear whether, in the face of repeated requests to report alcohol consumption, respondents simply report fewer drinks or have truly modified their drinking behavior (Clifford and Maisto 2000; Cliffort et al. 2007). It also is unclear whether the reported changes in drinking behavior are sufficient to change long-term clinical outcomes.

#### Biomarkers

Biomarkers provide the means to assess clinical effects of alcohol and aging directly and to prioritize these effects according to their likely cumulative effect on morbidity and mortality. The advantage of using biomarkers is that they may be able to provide information about important clinical changes before the changes are obvious to patients or health care providers. Therefore, biomarkers can serve as an “early warning system” in clinical trials and clinical care to help predict morbid events.

#### VACS Risk Index

VACS has recently developed an index of commonly available clinical lab values termed the VACS Risk Index (Justice et al. 2009). The VACS Risk Index is designed to be a prognostic index that reflects alcohol-, HIV-, and comorbid disease–related physiologic injury. The index includes indicators of liver injury, viral hepatitis, AIDS-defining illnesses (e.g., pneumocystis pneumonia, toxoplasmosis), renal injury, bone marrow injury, and immune suppression. The VACS Risk Index has demonstrated predictive accuracy for mortality among veterans in care with HIV infection on par with several established risk indices developed for other important clinical populations. For 30-day mortality, the VACS Index is as discriminating of mortality as the APACHE index is among intensive-care unit patients. It is more discriminating than the Charlson Comorbidity Index is for 1-year mortality among a general sample of individuals admitted to the hospital. Because it integrates immunologic virologic measures (i.e., CD4 cell count and viral load), disease measures (i.e., AIDS-defining conditions and viral hepatitis), and measures of organ system function (renal insufficiency, liver injury, and anemia), it reflects the multifaceted direct physiologic effects of alcohol and important interactions with viral hepatitis, HIV disease progression, and ART, including the resulting effects of poor adherence to ART. Most importantly, because the index focuses on biomarkers, it captures the summary biologic effect of alcohol on the individual rather than measuring standard units of exposure.

## VACS Plans for Strategy Implementation Trials

A multilevel, strategy intervention trial is required to address the many modifiable implications of alcohol consumption among those receiving treatment for HIV. Alcohol has systemic biologic effects and is associated with multiple high-risk behaviors, including other substance use, risky sex, and medication nonadherence. Levels of alcohol consumption not associated with harm among those without HIV infection may be harmful among infected individuals. Importantly, alcohol’s harmful effects are not likely to be uniform among HIV-infected individuals. Finally, patients who are not currently drinking may still be suffering long-term biomedical complications from past alcohol abuse or dependence. Therefore, it is important to address ongoing injury from other sources (e.g., medication toxicity, untreated chronic viral hepatitis). The measure of self-reported drinks per day does not capture any of these factors. By using the VACS index to estimate risk of mortality, to identify the degree to which alcohol represents a significant and modifiable risk of morbidity and mortality in our patients, and to act as a surrogate marker for morbid events, we can design more informative and effective intervention studies.

The authors propose to use the index as part of a fully integrated and staged behavioral, pharmacologic, and biomedical intervention designed to decrease alcohol use and minimize morbidity and mortality. The index will be used to characterize overall risk of mortality and proportion of risk attributable to alcohol. Changes in the index over time will be used as the primary outcome (integrated surrogate marker) of the study, and we will conduct a validation analysis of the association between self-reported measures of alcohol consumption and changes in the index. Thus, the index will be both an outcome and a means of motivating behavior change (e.g., decreasing or stopping alcohol consumption and improving medication adherence). The index also will provide a means of motivating providers to refer patients for more intensive alcohol treatment and to prioritize factors likely exacerbating effects of alcohol (e.g., HCV and HBV infection, depression, anemia, treatment toxicity).

Because organ system injury and mortality are accelerated among HIV patients, they provide an ideal population in which to test the use of a biomarker index. The authors are currently beginning feasibility studies and evaluating the discrimination of the index for important clinical outcomes (i.e., functional status, frailty, hospitalizations, quality of life) and it applicability in HIV-negative and non-veteran populations. Should this work prove successful, the approach may prove widely generalizable. The goal of the strategy implementation study will be to combine components that have individually demonstrated efficacy in order to maximize improvement in outcome. As such, we anticipate that these interventions will be focused on the science of implementation and demonstrating effectiveness among diverse populations of patients.

## Figures and Tables

**Figure:**
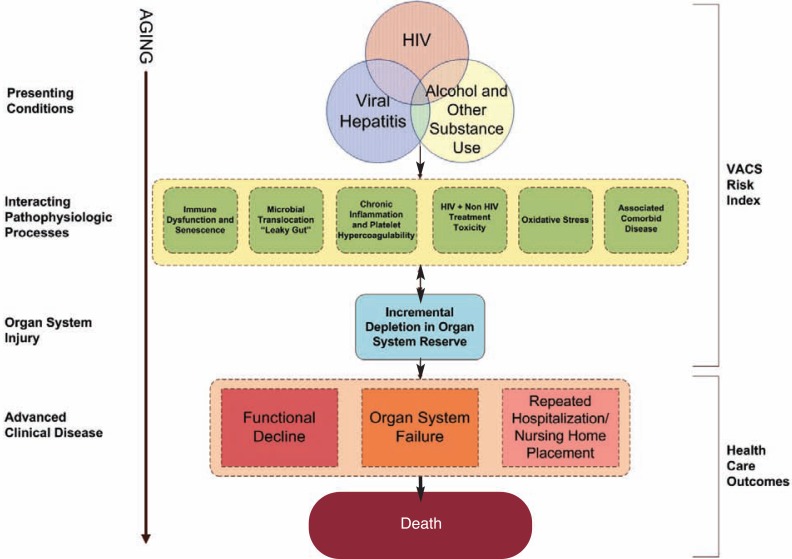
Conceptual model for aging with HIV infection. This figure illustrates the progression of disease among those in care who are aging with HIV infection from presenting health conditions through interacting disease processes to cumulative organ system injury, advanced clinical disease, and, eventually, death. The VACS Risk Index attempts to integrate and summarize the total disease process in order to better reflect risk of morbidity and mortality and to facilitate the identification and modification of risk.
